# Intracameral use of 2% lignocaine with preservative: Is it really safe?

**DOI:** 10.4103/0301-4738.58484

**Published:** 2010

**Authors:** Shivcharan L Chandravanshi, Sujata Lakhtakia, Mahesh K Rathore

**Affiliations:** Department of Ophthalmology, Shyam Shah Medical College, Rewa-486 001, M.P, India

Dear Editor,

We read with great interest the article by Gupta *et al*., on manual small incision cataract surgery (MSICS) under topical anesthesia with intracameral lignocaine.[1] The authors concluded that topical anesthesia using 2% lignocaine viscous and 0.5% intracameral lignocaine is safe and adequate for MSICS. We would like to raise a few issues for discussion.

- The authors have mentioned that sensitivity to lignocaine and inability to understand verbal commands were the only contraindications for topical anesthesia. However, certain patients who although can follow verbal commands, are either apprehensive or uncooperative and so may not be suitable candidates for topical anesthesia. Besides, patients with nystagmus would also be a contraindication to topical anesthesia.
- The authors have not mentioned the dose of intracameral lignocaine, whether it was given as a single injection or in divided doses and whether supplementary anesthetic eye drops were used during surgery. It is also mentioned in the study that 0.5% lignocaine with or without preservative is ‘safe’ for intracameral use. Regular 2% lignocaine and 2% lignocaine viscous contain methylparaben and propylparaben as preservatives. These preservatives have been shown to cause corneal endothelial toxicity in animal models although the effects are temporary. There is also no mention of pre- and postoperative endothelial cell count. Thus it would be premature to say that lignocaine with preservative is a ‘safe’ drug for intracameral injection without investigating this aspect.[2]
- In the present study, 2% lignocaine viscous was used after draping and was allowed to remain in contact with the ocular surface for about one minute after which surgery was commenced. It is not mentioned whether 2% lignocaine was washed before starting the surgical procedure or not and also whether one tube was used for single or multiple patients. Lignocaine 2% viscous can easily get contaminated if used on a shared basis thus posing a risk of postoperative endophthalmitis. Application of 5% povidone-iodine solution three times, five minutes apart after putting 2% lignocaine viscous and then thoroughly washing the conjunctival sac with sterile water before starting surgery, can reduce the risk of postoperative endophthalmitis and toxic anterior segment syndrome secondary to seepage of preservatives into the anterior chamber.[3,4]
- The authors have stated in their study that peribulbar anesthesia causes instability of anterior chamber secondary to raised intraocular pressure due to increased intraorbital volume. We feel that raised intraocular pressure is not responsible for anterior chamber instability since in the standard Blumenthal technique of MSICS, the intraocular pressure remains more than 35 mm of Hg throughout the procedure without compromising anterior chamber stability. On the contrary, constant lid squeezing, residual sensations and blepharospasm secondary to bright microscope light may be the causes for intraoperative anterior chamber instability rather then peribulbar anesthesia.[5]
